# Effect of routine vs on-demand nebulization of acetylcysteine with salbutamol on accumulation of airway secretions in endotracheal tubes: substudy of a randomized clinical trial

**DOI:** 10.1186/s40635-020-00351-x

**Published:** 2020-12-18

**Authors:** Sophia van der Hoeven, Lorenzo Ball, Federico Constantino, David M. van Meenen, Paolo Pelosi, Ludo F. Beenen, Marcus J. Schultz, Frederique Paulus, for the NEBULAE-investigators

**Affiliations:** 1 Department of Intensive Care, Amsterdam University Medical Centers, Location Academic Medical Center, Amsterdam, The Netherlands; 2 Department of Surgical Sciences and Integrated Diagnostics, University of Genoa, Genoa, Italy; 3 Anesthesia and Intensive Care, Ospedale Policlinico San Martino, IRCCS per l’Oncologia e le Neuroscienze, Genova, Italy, Genova, Italy; 4 Department of Anesthesiology, Amsterdam University Medical Centers, Location Academic Medical Center, Amsterdam, The Netherlands; 5 Department of Radiology and Nuclear Medicine, Amsterdam University Medical Centers, Location Academic Medical Center, Amsterdam, The Netherlands; 6 Laboratory of Experimental Intensive Care and Anesthesiology (L·E·I·C·A), Amsterdam University Medical Centers, Location Academic Medical Center, Amsterdam, The Netherlands; 7 Mahidol–Oxford Tropical Medicine Research Unit (MORU), Mahidol University, Bangkok, Thailand; 8 Nuffield Department of Medicine, University of Oxford, Oxford, UK; 9 ACHIEVE Centre of Applied Research, Faculty of Health, Amsterdam University of Applied Sciences, Amsterdam, The Netherlands

**Keywords:** Intensive care unit, Invasive ventilation, Nebulization, Acetylcysteine, Salbutamol, Airway secretions, Endotracheal tube

## Abstract

**Background:**

Accumulated airway secretions in the endotracheal tube increase work of breathing and may favor airway colonization eventually leading to pneumonia. The aim of this preplanned substudy of the ‘Preventive Nebulization of Mucolytic Agents and Bronchodilating Drugs in Intubated and Ventilated Intensive Care Unit Patients trial’ (NEBULAE) was to compare the effect of routine vs on-demand nebulization of acetylcysteine with salbutamol on accumulation of secretions in endotracheal tubes in critically ill patients.

**Results:**

In this single-center substudy of a national multicenter trial, patients were randomized to a strategy of routine nebulizations of acetylcysteine with salbutamol every 6 h until end of invasive ventilation, or to a strategy with on-demand nebulizations of acetylcysteine or salbutamol applied on strict clinical indications only. The primary endpoint, the maximum reduction in cross-sectional area (CSA) of the endotracheal tube was assessed with high-resolution computed tomography. Endotracheal tubes were collected from 72 patients, 36 from patients randomized to the routine nebulization strategy and 36 of patients randomized to the on-demand nebulization strategy. The maximum cross-sectional area (CSA) of the endotracheal tube was median 12 [6 to 15]% in tubes obtained from patients in the routine nebulization group, not different from median 9 [6 to 14]% in tubes obtained from patients in the on-demand nebulization group (*P* = 0.33).

**Conclusion:**

In adult critically ill patients under invasive ventilation, routine nebulization of mucolytics and bronchodilators did not affect accumulation of airway secretions in the endotracheal tube.

*Trial registration* Clinicaltrials.gov Identifier: NCT02159196

## Introduction

Intensive care unit (ICU) patients frequently need invasive ventilation, which is associated with retention of airway secretions that ultimately may accumulate in the larger airways and in the endotracheal tube. This increases airway resistance and thus work of breathing [1, 2]; furthermore, accumulated secretions serve as a substrate to the formation of bacterial biofilms, contributing to the risk of ventilator-associated pneumonia [3–5].

Nebulization of mucolytic agents with bronchodilating drugs is a strategy aiming at prevention of airway obstruction in critically ill patients under invasive ventilation. As it was uncertain whether such nebulizations should be applied routinely or only on strict clinical indications, the recently published ‘Preventive Nebulization of Mucolytic Agents and Bronchodilating Drugs in Intubated and Ventilated Intensive Care Unit Patients trial’ (NEBULAE) was performed. This randomized clinical trial showed an on-demand strategy of nebulizations of acetylcysteine or salbutamol not to be inferior to a routine strategy of nebulizations of acetylcysteine with salbutamol with respect to the number of ventilator-free days at day 28 [6].

The aim of the current substudy of NEBULAE was to compare the routine nebulization strategy with the on-demand nebulization strategy with respect to accumulations of airway secretions in the endotracheal tube. In line with the hypothesis of the parent trial, it was hypothesized that a strategy of on-demand nebulizations of mucolytics or bronchodilators would not be inferior to a strategy of routine nebulizations of mucolytics with bronchodilators with respect to reduction in cross-sectional area (CSA) of the endotracheal tube.

## Materials and methods

### Design and ethical approval

This was a preplanned single-center substudy of NEBULAE [6], a national multicenter randomized clinical trial in the Netherlands. This substudy took place in the ICU of the Amsterdam University Medical Centers, location Academic Medical Center, Amsterdam, The Netherlands. The study design of the parent study was prepublished [7], and the results of the primary analysis were published upon termination of the study [6]. The Institutional Review Board of the AMC approved both the protocol of the parent study, and also this preplanned substudy (2014_088). Written informed consent was obtained before participation in the parent study; collection of endotracheal tubes after tracheal extubations did not require additional informed consent.

### Patients

The parent study enrolled patients under invasive ventilation who were expected to not be extubated within 24 h after randomization. Exclusion criteria included age younger than 18 years; pregnancy; ventilation lasting more than 24 h before randomization; known allergy against study medication, or a history mandating continuation of mucolytic agents or bronchodilating drugs during invasive ventilation. The current substudy used the same inclusion and exclusion criteria, but as this study could only ran in the AMC, the single additional inclusion criterion was that a patient needed to be admitted to the ICU of this hospital.

### Intervention

Patients were randomized in a 1:1 ratio to a strategy of routine nebulizations of acetylcysteine with salbutamol (the ‘routine nebulization strategy’) or one of nebulizations of acetylcysteine or salbutamol on strict clinical indications (the ‘on-demand nebulization strategy’). Patients assigned to the routine nebulization strategy received nebulization of 5–ml solutions containing 300 mg acetylcysteine with 5-ml solutions containing 2.5 mg salbutamol four times daily, from start to end of invasive ventilation. Patients assigned to the on-demand nebulization strategy could receive nebulization of acetylcysteine when thick or tenacious secretions were noted or nebulization of salbutamol when wheezing was clinically suspected or observed, or when typical abnormalities of ventilator waves or end-tidal CO_2_ curves suggested obstruction of the lower airways. The continued need for nebulization of acetylcysteine or salbutamol was reassessed daily by independent physicians.

### Standard care

Standard care followed local clinical guidelines and was performed by independent board-certified ICU physicians and board-certified ICU nurses not involved in the trial. Physician and nursing staffs were encouraged to use lung-protective ventilation strategies including the use of low tidal volumes and positive end-expiratory pressures adjusted to severity of lung disease, restrictive sedation preferring analgosedation over hypnosedation, and an intravascular fluid protocol preferring a restrictive over a liberal fluid strategy. All patients received selective decontamination of the digestive tract. ICU nurses performed standard airway care, including endotracheal suctioning, and humidification of inhaled air if applicable. Endotracheal suction was performed only when clinically indicated and according to current guidelines. Humidification of the inhaled air was guaranteed by using heat and moister exchanger-filters.

### Tube sampling process

**Fig. 1:**
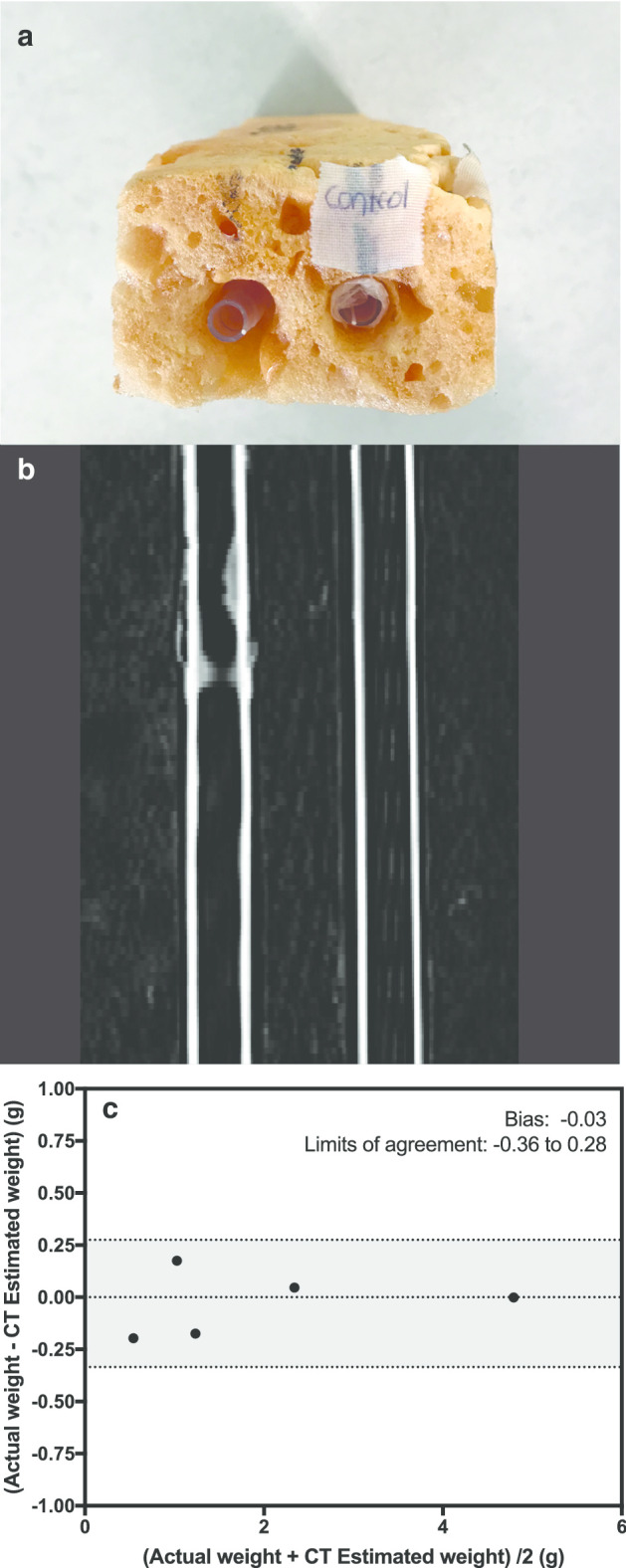
Tube mold used for image acquisition (**a**) and corresponding computed tomography image (**b**) of a partially obstructed tube (left) and the control tube (right). Bland–Altman analysis between CT-estimated and scale-measured mucus weight using a threshold of − 770 HU (**c**). CT, computed tomography.

Endotracheal tubes were collected after tracheal extubation and sealed at the extremities with a plastic film to prevent drying of secretions. Tubes were carefully stored in a horizontal position at 4 °C to preserve secretion configurations. Tube scanning was performed as soon as possible but always within 72 h after tracheal extubation. High-resolution CT images were obtained using a Siemens Sensation 64 scanner (Siemens Healthcare, Forchheim, Germany; scan parameters 100Kvp, 100 mAs) adapting a method previously described [2, 8]. Tubes were scanned, for a length of 25 cm from the tip while inserted in the polyurethane foam mold along with a clean tube of the corresponding size as a control (Fig. 1).

### Tube image analysis

Images were reconstructed in 1-mm slices with a sharp B70 convolution kernel, then automatically segmented and analyzed with an operator-independent dedicated software written in MATLAB (MathWorks Inc. US). Distinction between the luminal space open to ventilation and secretions was based upon differences in density (HU). For each CT slice, the inner lumen of the test and control tubes were identified with an automated algorithm based on the circular Hough transform. The surface occupied by mucus was computed as the difference, between the test and control tubes, of the voxels above a certain Hounsfield Units (HU) threshold.

The cross-sectional area (CSA) percent reduction was then calculated as the difference between effective CSA in the patient’s tube and the corresponding control tube, divided by the CSA of the control tube.

Feasibility and validation of the CSA assessment method have been tested in a bench-top study, readapting the method used by Coppadoro et al. [8]. For this calibration procedure five different known amounts of a water-based polymeric gel have been injected into clean endotracheal tubes (internal diameter 8.0 mm). The exact amount of injected gel has been measured weighing the endotracheal tube with a precision scale before and after gel injection. Like in the patients’ tubes, the calibration tubes were inserted in the scanning box along with a clean control tube. Images were acquired and analyzed with the same settings as the patients’ tubes. The threshold has been chosen as the HU cut-off in the maximum Pearson’s correlation coefficient between weights estimated with the CT analysis and those obtained with the gravimetric scale in an in vitro calibration procedure. We tested all possible HU cut-offs between − 900 and − 100 HU in 5-HU steps and observed the best correlation with threshold set at − 770 HU. At this threshold, we performed a Bland–Altman comparison between gravimetric scale in an in vitro calibration procedure (Fig. 1). It was assumed that *X* grams (or X ml) of gel would homogeneously distribute inside a 20-cm section of a 8-mm tube. As this tube has an inner volume of around 10 ml, this translates to a *X*/10*100% volume reduction, or, at the slice level, a *X*/10*100% CSA reduction. Under these assumptions, the Bland–Altman analysis translated to the CSA% level would correspond to a bias of − 0.3% and a 95% limit of agreement from − 3.6% to + 2.8%.

### Outcome

The primary outcome was the amount of endotracheal tube occlusion, visualized by means of the lowest CSA of the tube available for ventilation.

As secondary endpoint, we also observed the distance between the tip of the tube and the point where the maximum obstruction, i.e., the highest proportional reduction in CSA reduction, was present in the endotracheal tube.

### Statistical analysis

Based on previous observational studies using a similar technique for evaluation of endotracheal tube occlusion, a maximum CSA reduction of 25 ± 4% was expected [2, 8, 9]. We had no preliminary data concerning how nebulization could affect CSA reduction by secretions. Therefore, we arbitrarily considered a clinically relevant reduction of CSA from 25 to 21%, which corresponds to a relative reduction of secretions amount of around 20%. A sample consisting of at least 26 patients for each arm (total 52 patients) would have a 90% power (1 − *β*) to detect a CSA reduction from 25 to 21% (effect size *d* = 1), with an alpha level of 0.05. Sample size calculation was performed with G*Power 3 [10]

The primary analysis was performed according to a modified intention-to-treat principle, by randomization group, and used a multilevel linear regression model to compare the maximum CSA reduction between the randomization groups with a two-sided 95% confidence interval (CI). To assess the association between CSA and patient variables multivariate regression analysis was used. An etiological model with a forced-entry strategy was chosen. Six variables (randomization group, duration of mechanical ventilation, reason for mechanical ventilation, gender, APACHE II, and tube diameter) assumed to have an association with CSA reduction were selected.

Continuous variables were expressed by their median with interquartile ranges. Categorical variables were expressed as *n* (%). Tests between groups Student’s *t*-test was used with *α* = 0.05 and with 2-sided 95% CIs, for continuous data that were not normally distributed the Mann–Whitney *U* test was used. Categorical variables were compared with the Chi-square test or Fisher’s exact tests. Mucus distribution was compared with a two-way ANOVA using the distance from the tube tip and the randomization arm as factors.

All statistical analyses were performed in R (R Core Team, 2016) software statistics version 3.4.3 [11]. A *P*-value of 0.05 indicated a statistically significant difference.

## Results

### Patients

**Fig. 2:**
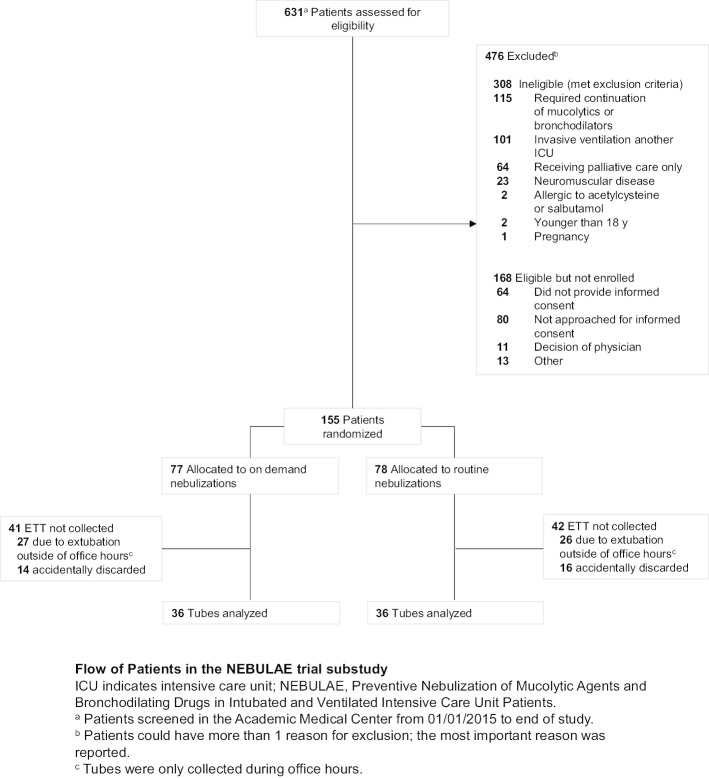
Flow of patients in the effect of on-demand vs routine nebulization of acetylcysteine with salbutamol on endotracheal tube occlusion assessed with computed tomography a substudy of the NEBULAE randomized trial. ICU, intensive care unit; ETT, endotracheal tube

Patient flow is presented in Fig. 2. From January 1 2015 to November 24 2016, a total of 631 patients were screened, of which 155 were included in the parent study in the center were this substudy ran. From 72 consecutive patients the endotracheal tube could be collected, from 36 patients randomized to the on-demand nebulization group and from 36 patients randomized to the routine nebulization group.

Patient characteristics at baseline are shown in Table 1. Baseline characteristics were comparable between the two randomization groups, and were neither different between patients included in the parent study and patients included in this substudy, nor between patients from whom the endotracheal tube could or could not be collected.

**Table 1:** Baseline characteristics, and main outcome of patients receiving on-demand nebulizations vs. routine nebulizations

Baseline characteristic	On-demand nebulization group (*n* = 36)	Routine nebulization group (*n* = 36)
Age, median (IQR), years	65 [59–73]	61 [51–69]
Women, no. (%)	11 (31)	12 (33)
BMI, median (IQR), kg/m^2^	25 [23 -29]	25 [23–28]
APACHE II, median (IQR)	20 [10—22]	17 [10–20]
Reason of ICU admission, no. (%)		
Medical	24 (67)	23(64)
Surgical	12 (33)	13 (36)
Reason of invasive ventilation, no. (%)		
OHCA	5 (14)	4 (11)
Postoperative ventilation	6 (17)	3 (8)
Head trauma or brain surgery	7 (19)	4 (11)
Pneumonia	3 (8)	1 (3)
Sepsis	1 (3)	4 (11)
Cardiac failure	1 (3)	4 (11)
Trauma	4 (11)	8 (22)
Aspiration	1 (3)	2 (6)
ARDS	0 (0)	1 (3)
Respiratory insufficiency	8(22)	5 (14)
Comorbidity, no, (%)		
Diabetes mellitus	9 (25)	4 (11)
Cardio-vascular disease	7 (19)	8 (22)
Pulmonary disease	3 (8)	3 (8)
Immunosuppression	8 (22)	3 (8)
Outcome data		
Exposure of ETT to invasive ventilation, median (IQR), days*	6 [2]	5 [2–14]

IQR, interquartile range; BMI, body mass index; APACHE, Acute Physiology and Chronic Health Evaluation; ARDS, acute respiratory distress syndrome; OHCA, out of hospital cardiac arrest; ETT, endotracheal tube

In the routine nebulization group, 35 patients (97%) received nebulizations, totaling 384 nebulization days. One patient in the routine nebulization group did not receive any nebulization according to the study protocol due to an unexpected short duration of ventilation. In the on-demand nebulization group, 19 patients (53%) received nebulizations, totaling 67 nebulization days. The total number of nebulizations received was a median of 27 [12 to 40] for both acetylcysteine and salbutamol for the patients in the routine nebulization group versus 1 [0 to 2] for acetylcysteine and 0 [0 to 0] for salbutamol for the 36 patients in the on-demand nebulization group. There was neither a difference in the number of endotracheal suctioning procedures per day, nor the number of artificial coughs by manual hyperinflation per day, between the two randomization groups (Table 2).

**Table 2:** Respiratory management of patients receiving demand nebulizations vs. routine nebulizations

	On-demand nebulization group	Routine nebulization group
Nebulization, no. per day, median (IQR)		
Acetylcysteine	0 [0–0]	4 [4–4]
Salbutamol	0 [0–0]	4 [4–4]
Nebulization, total amount, median (IQR)		
Acetylcysteine	1 [0–2]	27 [12–40]
Salbutamol	0 [0–0]	27[12–40]
Nebulization, no. of days with, median (IQR)		
Acetylcysteine	1 [0–2]	6 [3–15]
Salbutamol	0 [0–0]	6 [3–15]
Endotracheal suctioning, no. per day, median (IQR)	5 [4, 8]	6 [4, 10]
Manual hyperinflation, no. per day, median (IQR)	3 [2, 3]	3 [2–4]

IQR, interquartile range

### Accumulations of sputum in endotracheal tubes

**Fig. 3:**
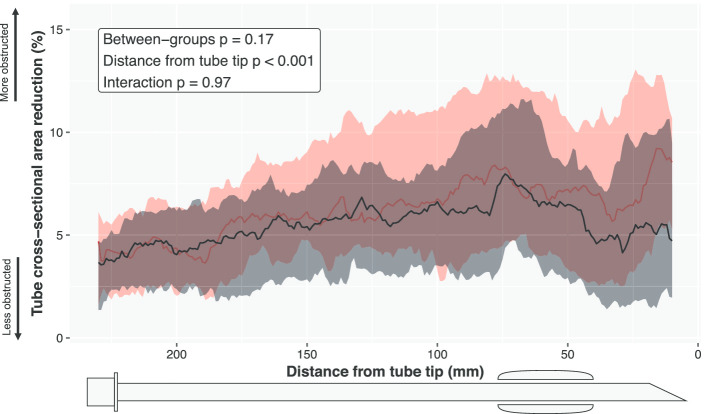
Endotracheal tube percent cross-sectional area reduction of each 1-mm section as function of the distance from the tube tip in patients receiving on-demand nebulizations (black) and patients receiving routine nebulizations (red). Values are plotted as median; error bands represent interquartile ranges. This plot represents the average mucus distribution in the two randomization arms, which is lower compared to the maximum cross-sectional area reduction of each patient. *P*-values are calculated with a two-way ANOVA using the distance from the tube tip and the randomization arm as factors. mm, millimeter

All endotracheal tubes were scanned and analyzed according to the study protocol. Maximum CSA reduction was not affected by the nebulization strategy: 12 [6 to 15]% versus 9 [6 to 14]% in the routine nebulization group and the on-demand nebulization group, respectively (*P* = 0.33). The distance from the tube tip to the location where CSA was smallest was also not different, 42 [14–89] mm versus 60 [10–88] mm in the routine nebulization group and in the on-demand nebulization group, respectively (*P* = 0.86) (Fig. 3).

Univariate and multivariate analysis did not show statistically significant associations between CSA and any of the analyzed patient variables (Table 3).

**Table 3:** Association between CSA and patient variables

Variable	Univariate	*P* value	Multivariate	*P* value
	estimate with 95% CI		estimate with 95% CI	
Randomization group	− 1.1 (− 5.1 to 3.0)	0.60	1.3 (− 4.6 to 7.3)	0.65
Duration of mechanical ventilation	− 0.2 (− 0.5 to 0.1)	0.21	− 0.1 (− 0.6 to 0.3)	0.56
Reason for mechanical ventilation (direct pulmonary vs. indirect pulmonary)	1.6 (− 3.6 to 6.9)	0.54	5.1 (− 3.5 to 13.7)	0.24
Gender	2.8 (− 1.5 to 7.1)	0.19	3.1 (− 4.0 to 10.3)	0.39
APACHE II	− 0.2 (− 0.5 to 0.2)	0.43	− 0.2 (− 0.7 to 0.2)	0.35
Tube diameter	− 1.2 (− 5.4 to 3.0)	0.58	0.5 (− 6.9 to 7.9)	0.89
Age	− 0.0 (− 0.2 to 0.1)	0.71		
History of diabetes	0.5 (− 4.7 to 5.8)	0.84		
History of CVD	− 2.0 (− 6.9 to 3.0)	0.42		
History of pulmonary disease	1.6 (− 5.7 to 8.9)	0.66		
BMI	− 0.0 (− 0.4 to 0.3)	0.98		
Total amount of acetylcysteine nebulizations	− 0.0 (− 0.1 to 0.1)	0.77		
Total amount of salbutamol nebulizations	− 0.0 (− 0.1 to 0.1)	0.91		
Acetylcysteine nebulizations per day	− 0.1 (− 0.5 to 0.3)	0.64		
Salbutamol nebulizations per day	− 0.0 (− 0.4 to 0.3)	0.81		
Total amount of nebulizations per day	− 0.1 (− 0.5 to 0.3)	0.69		
Amount of acetylcysteine nebulization days	− 0.0 (− 0.3 to 0.3)	0.89		
Amount of salbutamol nebulization days	0.0 (− 0.3 to 0.3)	0.95		
Nebulizations days	− 0.0 (− 0.3 to 0.3)	0.97		
Total amount of nebulizations	− 0.0 (− 0.1 to 0.1)	0.79		

CI, confidence interval; APACHE II, Acute Physiology and Chronic Health Evaluation II; CVD, cardio-vascular disease; BMI, body mass index

## Discussion

This is the first study that assessed the effects of two different nebulization strategies on accumulation of airway secretions in the endotracheal tube in adult critically ill patients under invasive ventilation. The findings of this study can be summarized as follows: (1) accumulation of airway secretions are not different between the two nebulization strategies; and (2) also the location of accumulated secretions is not different dependent on the nebulization strategies. This preplanned substudy of NEBULAE aims to strengthen the knowledge about effect of nebulization on mucus plugging in intubated patients in ICU. The current findings are in line with those in the parent study trial [6].

The parent study had several strengths. Bias was controlled by using concealed allocation and intention-to-treat analysis using robust protocols, loss to follow-up was minimal and the trial involved 7 centers, contributing to its generalizability. The on-demand strategy was designed to receive the minimal number of nebulizations per patient and according to clinical needs. We also deliberately chose not to combine the acetylcysteine and salbutamol in the on-demand group. Care for patients was standardized using clinical protocols and nurses were skilled in nebulization therapy, and last but not least patients were enrolled over a period of 2 years during which care had not changed. This substudy also had strengths. First, the substudy was preplanned, and prospective in design. Second, a validated technique and a robust protocol were used to investigate the effects of nebulization on the CSA of the endotracheal tubes [8].

The findings of this study are in line with those from previous investigations in distribution of airway secretions in endotracheal tubes [2, 8]. For instance, we here observed an identical longitudinal CSA distribution along the tube, with a progressively decreasing CSA from the proximal to the distal end of the endotracheal tube [2, 8].

One possible explanation for the neutral findings of this substudy shares the same hypothesis as the parent study, that is that prevention of mucus plugging with on-demand nebulizations is noninferior to that obtained with routine nebulization. Another possible hypothesis is that the standard airway care management of ICU patients is sufficient to minimize the role of nebulization.

Several limitations should be noted. First, the sample size of this substudy was small and we were not able to collect all the endotracheal of patients included in the parent study in the center where this substudy ran. Second, the lack of blinding could have influenced nurses behavior in this study. However, patients in the on-demand nebulization group did not receive more additional airway care compared to patients in the routine nebulization group. Finally, we compared ex vivo images of the endotracheal tubes, in which tubes were examined in a straight horizontal position. In vivo endotracheal tubes are typically curved, and moreover they tend to bend or kink in oral cavity and in the throat, as such increasing the risk of subtotal or even total tube occlusion. However, this effect, probably is the same as the sputum accumulations were not different between the two study groups.

## Conclusion

In conclusion, the two compared strategies of nebulization did neither affect amount nor the locations of accumulated airway secretions in endotracheal tubes of ICU patients. These findings support the findings of the parent study that showed an on-demand strategy of nebulizations of acetylcysteine or salbutamol not to be inferior to a routine strategy of nebulizations of acetylcysteine with salbutamol with regard to clinical endpoints.

## Data Availability

The datasets used and/or analyzed during the current study are available from the corresponding author on reasonable request.

## Abbreviations

AMC: Academic Medical Center
°C: Degree Celsius
CI: Confidence interval
cm: Centimeter
CO_2_: Carbon dioxide
CSA: Cross-sectional area
CT: Computed tomography
HU: Hounsfield units
ICU: Intensive care unit
mg: Milligram
ml: Milliliter
mm: Millimeter

## References

[1] Shah C, Kollef MH (2004). Endotracheal tube intraluminal volume loss among mechanically ventilated patients. Crit Care Med.

[2] Mietto C, Pinciroli R, Piriyapatsom A, Thomas JG, Bry L, Delaney ML (2014). Tracheal tube obstruction in mechanically ventilated patients assessed by high-resolution computed tomography. Anesthesiology.

[3] Vandecandelaere I, Coenye T (2015). Microbial composition and antibiotic resistance of biofilms recovered from endotracheal tubes of mechanically ventilated patients. Adv Exp Med Biol.

[4] Sottile FD, Marrie TJ, Prough DS, Hobgood CD, Gower DJ, Webb LX (1986). Nosocomial pulmonary infection: possible etiologic significance of bacterial adhesion to endotracheal tubes. Crit Care Med.

[5] Donlan RM, Costerton JW (2002). Biofilms: survival mechanisms of clinically relevant microorganisms. Clin Microbiol Rev.

[6] van Meenen DMP, van der Hoeven SM, Binnekade JM, de Borgie C, Merkus MP, Bosch FH (2018). Effect of on-demand vs routine nebulization of acetylcysteine with salbutamol on ventilator-free days in intensive care unit patients receiving invasive ventilation: a randomized clinical trial. JAMA.

[7] van der Hoeven SM, Binnekade JM, de Borgie CA, Bosch FH, Endeman H, Horn J (2015). Preventive nebulization of mucolytic agents and bronchodilating drugs in invasively ventilated intensive care unit patients (NEBULAE): study protocol for a randomized controlled trial. Trials.

[8] Coppadoro A, Bellani G, Bronco A, Borsa R, Lucchini A, Bramati S (2014). Measurement of endotracheal tube secretions volume by micro computed tomography (MicroCT) scan: an experimental and clinical study. BMC Anesthesiol.

[9] Pinciroli R, Mietto C, Berra L (2013). Use of high-definition computed tomography to assess endotracheal tube luminal narrowing after mechanical ventilation. Anesthesiology.

[10] Faul F, Erdfelder E, Lang AG, Buchner A (2007). G*Power 3: a flexible statistical power analysis program for the social, behavioral, and biomedical sciences. Behav Res Methods.

[11] Team RC (2018). R: A language and environment for statistical computing.

